# Systematic Analysis of the Relative Abundance of Polymers Occurring as Microplastics in Freshwaters and Estuaries

**DOI:** 10.3390/ijerph17249304

**Published:** 2020-12-12

**Authors:** John Iwan Jones, Alena Vdovchenko, Dave Cooling, John F. Murphy, Amanda Arnold, James Lawrence Pretty, Kate L. Spencer, Adriaan Albert Markus, A. Dick Vethaak, Marina Resmini

**Affiliations:** 1 Department of Biology, School of Biological and Chemical Science, Queen Mary University of London, London E1 4N, UK; Dave.cooling@me.com (D.C.); j.f.murphy@qmul.ac.uk (J.F.M.); a.arnold@qmul.ac.uk (A.A.); j.l.pretty@qmul.ac.uk (J.L.P.); 2 Department of Chemistry, School of Biological and Chemical Science, Queen Mary University of London, London E1 4NS, UK; a.vdovchenko@qmul.ac.uk; 3 School of Geography, Queen Mary University of London, London E1 4NS, UK; k.spencer@qmul.ac.uk; 4 Deltares, Marine and Coastal System, Boussinesqweg 1, 2629 HV Delft, The Netherlands; Arjen.Markus@deltares.nl (A.A.M.); Dick.Vethaak@deltares.nl (A.D.V.); 5 Department of Environment and Health, Vrije Universiteit Amsterdam, 1081 HV Amsterdam, The Netherlands

**Keywords:** microplastic, relative abundance, environmental impact, plastic polymers

## Abstract

Despite growing interest in the environmental impact of microplastics, a standardized characterization method is not available. We carried out a systematic analysis of reliable global data detailing the relative abundance of polymers in freshwaters and estuaries. The polymers were identified according to seven main categories: polyethylene terephthalate, polyethylene, polyvinyl chloride, polypropylene, polystyrene, polyurethane and a final category of miscellaneous plastic. The results show that microplastics comprised of polyvinyl chloride and polyurethane are significantly less abundant than would be expected based on global production, possibly due to their use. This has implications for models of microplastic release into the environment based on production and fate. When analysed by matrix (water, sediment or biota) distinct profiles were obtained for each category. Polyethylene, polypropylene and polystyrene were more abundant in sediment than in biota, while miscellaneous plastics was more frequent in biota. The data suggest that environmental sorting of microplastic particles, influenced by physical, chemical and biological processes, may play a key role in environmental impact, although partitioning among matrices based on density was not realized. The distinct profile of microplastics in biota raises an important question regarding potential selectivity in uptake by organisms, highlighting the priority for more and better-informed laboratory exposure studies.

## 1. Introduction

Plastics are synthetic polymers which can be made into a vast range of inexpensive, lightweight and durable products, ranging from single use products, such as plastic bags and food wrappings, to reusable items and products, such as building materials expected to have a lifetime of around 35 to 40 years. The chemical structure and formulations of these materials, together with their manufacturing processes are responsible for their specific properties, including durability and resistance to various environmental agents, such as heat, pH, and UV and IR radiation [1]. Over the last fifty years, the plastic industry has grown exponentially to reach a global production of 359 million tonnes of plastics per annum (data from 2018 [2]). Whilst the majority of post-consumer plastic waste is collected and re-used (recycling/energy recovery) or disposed of via landfill [2], a substantial proportion is mismanaged and may be released into the environment [3]. The impact, behaviour and fate of plastic polymers in the environment is the subject of global concern and debate: the durability and robustness of these materials is correlated with their persistence in the ecosystem [4,5,6].

It is now recognized that microsized particles of plastic, named microplastics, are present in the environment [7]. The European Chemical Agency defines these microplastics as any polymer, or polymer-containing solid or semi-solid particle having a maximum size of 5mm or less in their largest dimension [8]. This includes both microplastics produced on purpose (i.e., primary microplastic, e.g., cosmetic exfoliates, air blasting media) and those formed through the breakdown of larger particles discarded or in use (i.e., secondary microplastic). Given that the amount of total plastic discarded globally has been estimated to reach 12 billion tonnes by 2050 [9], the amount of microplastic released in the environment is also expected to grow. It has been suggested that microplastic particles have a significant impact on biota, resulting in associated costs predicted to be over $13 billion each year [10].

As plastics are manmade; the composition of microplastics in the environment will reflect the different types of polymers used. Polyethylene terephthalate (PET), polypropylene (PP), low- and high-density polyethylene (LDPE and HDPE), polyvinyl chloride (PVC), polystyrene (PS) and polyurethane (PU) are the main polymers based on production volumes [2], although a wide array of polymers are produced commercially, including among others polyamide, polymethyl methacrylate, bioplastics and polyethylene-vinyl acetate. The different chemistry of each plastic, together with the production process and its varied use in products (on its own or in combination with others) influences the rate of polymer degradation and, therefore, the amount of microplastic generated and released into the environment. In addition, as with all particles moving through the environment, various physically, chemically and biologically related processes may influence the degradation and transport of plastic particles [11,12], resulting in a substantial variation in the concentration profiles of microplastics, both in time and space. As the transport of particles is heavily influenced by the flow of water through the landscape [13], rivers have been highlighted as being an important pathway for the transport of microplastics [14,15] and, hence, potentially being particularly vulnerable to any impact they may cause. Furthermore, as the management of plastic pollution needs to be informed regarding the sources delivering microplastics into the environment, there is a need to better understand all aspects of phenomena associated with microplastic pollution, and more specifically to gather evidence of the prevalence of different polymers in freshwaters and estuaries.

Despite considerable recent interest in microplastics in freshwaters and estuaries, the scientific community has yet to agree on a standardized method for the sampling and analysis of microplastics in environmental samples. In addition, there are considerable challenges in the identification of the sources contributing to the load of microplastics in rivers and estuaries. Typically, there are few characteristics other than the plastic polymer type on which to attribute source. In the absence of this information, policy to reduce the impact of plastic pollution has been developed using models of use and assumed release of microplastics into the environment, which are yet to be verified. As such, the profile of polymers found in rivers and estuaries is of keen interest.

In this work, we present the first systematic analysis of global data on the relative abundance of microplastics in freshwaters and estuaries. We focused on the polymers reliably identified from the microplastics found in freshwater and estuarine environments and compared these with reported production. To enable comparison across different data sources, we used relative abundance (percentage by polymer). The data were then further analysed to determine if there were differences in the relative abundance of microplastic polymers identified in water, sediments and biota. Such differences would inform how transport and degradation processes may influence the movement of microplastics through the environment and, hence, the exposure of biota to any potential adverse effects.

## 2. Materials and Methods

### 2.1. Evidence Capture

Data on the prevalence of microplastics in freshwaters and estuaries were compiled from the literature, including grey literature, published up until April 2019 using the systematic review procedure [16]. As microplastics have only been studied recently [7], no earliest date was used to define the date range of publications included. An exception on the date range was made for two relevant reports [17,18]. To establish the wider population of evidence on microplastics in freshwaters and estuaries, the following search terms were used (with BOOLEAN operators): microplastic, nanoplastic, freshwater, estuary*, transitional, ecosystem, aquatic, wetland, river, stream, brook, lake, pool, pond, reservoir, marsh, swamp, aquifer, groundwater, wastewater, sewage, outfall, potable, and drinking water. Searches were made using BioOne, COPAC, DART-Europe E-theses Portal, EBSCO Open dissertations, EThOS: Electronic Theses Online Service, European Commission Research Publications, European Sources Online, GoogleScholar, MedLine, JStor, SciFinder, Open Access Theses and Dissertations, OpenGrey, PubMed, PLoS, Scopus, SciFinder, Web of Science and holdings of relevant environmental regulators.

The overall evidence base on microplastics in freshwaters and estuaries identified 3456 unique sources, the details of which were retained in a searchable database. This population of evidence was searched further using search terms to further refine the population of evidence and identify evidence relevant to the quantification and characterisation of microplastics in freshwaters and estuaries (Table 1). There were no comparator elements in this search.

Evidence sources were further screened by a team of reviewers to remove evidence sources not relevant to freshwaters or estuaries, to identify reviews (which were used for further identification of evidence sources, but not included in data capture unless novel data were presented) and duplicate information, and identify sources likely to contain relevant evidence.

Data were captured from evidence sources by the reviewers, according to pre-defined information fields relevant to the key question and sub-questions being addressed. In total, 209 unique evidence sources provided information on the quantification and characterisation of microplastics in freshwaters and estuaries.

Data extracted from evidence sources included details of the environment and matrix sampled, the sampling procedure, the separation and quantification technique(s) used, and the polymers identified. Data were captured separately where evidence sources presented results obtained by different sampling methods or different matrices.

**Figure 1:**
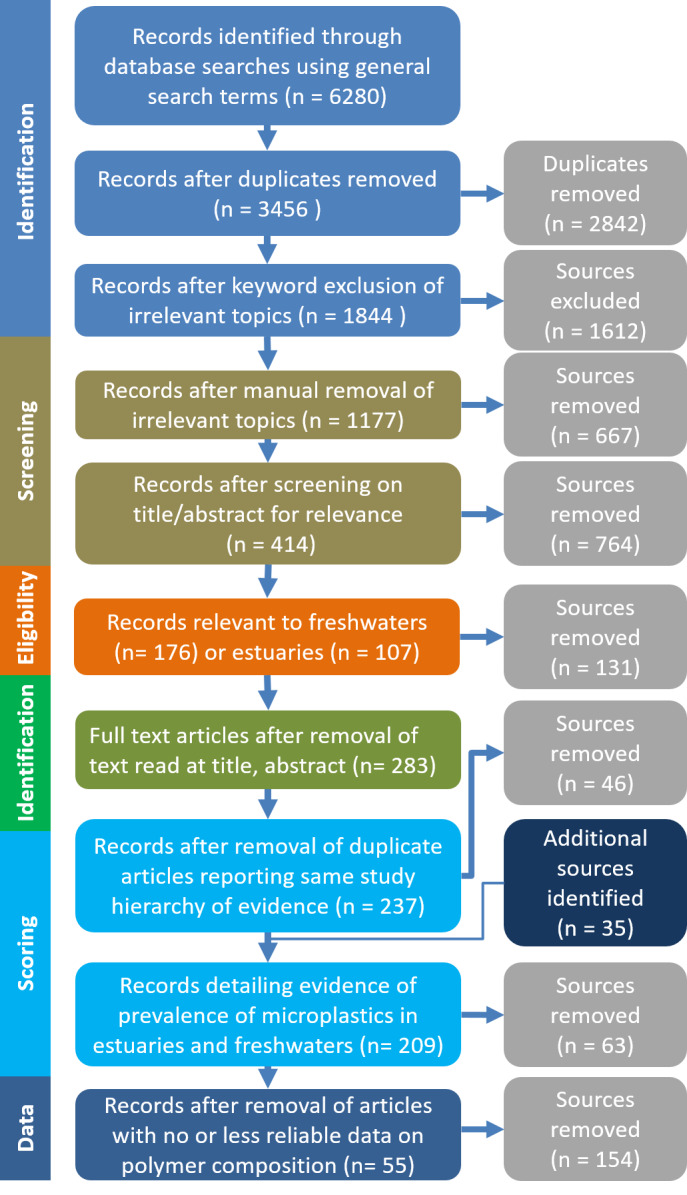
Map of evidence identified as relevant to this systematic review.

Following the methods of Hermsen et al. [19] and Koelmans et al. [20], the reliability of the evidence describing the relative abundance of polymers was assessed, and only evidence judged to be reliable without restrictions was used. This comprised only those evidence sources where polymers were identified using FTIR, Raman or GCMS on at least a representative subsample of ≥ 50 particles or ≥ 25% of the filter area. After this final screening process, 55 unique evidence sources were retained, which provided 71 measures of the composition of microplastics in environmental samples (Figure 1).

### 2.2. Statistical Analyses

Paired t-tests (with Bonferroni corrected acceptance levels) were used to determine if the relative abundance of polymers described from environmental samples were significantly different to that expected from relative global production [2], waste production [9] or from polymer-specific modelled release of microplastics into surface waters [3]. To ensure normality, all data were arcsine transformed before use. MANOVA (multivariate analysis of variance) was used in SAS/STAT^®^ (SAS Institute Inc., Cary, North Carolina, USA) to determine if the relative abundance of polymers described differed among the three matrices, water, sediment and biota, with Tukey’s post hoc tests being used to identify where significant differences lay within polymers. Again, all data were arcsine transformed before use.

## 3. Results

**Figure 2:**
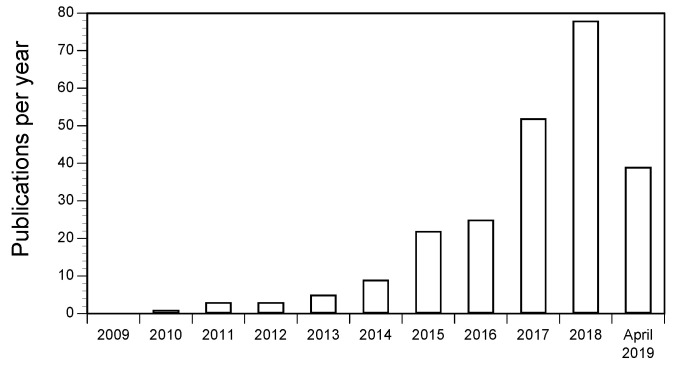
Number of publications detailing the quantification and characterisation of microplastics in freshwaters and estuaries per year. For 2019, only data up to April are included in this review.

The systematic review procedure [16] identified 209 unique evidence sources detailing the quantification and characterisation of microplastics in freshwaters and estuaries (Figure 1). It was evident that the number of studies published investigating microplastics in freshwaters and estuaries has increased exponentially in recent years (Figure 2). Yet, to date there has been no consensus on the methods used to sample and analyse microplastics. We used a generally accepted a priori review method [17,21] to identify those data that were based on methods that are considered reliable without restrictions with regard to the characterisation of the polymers found. Using this approach, 55 unique evidence sources provided reliable evidence of the relative abundance of polymers found as microplastics in rivers and estuaries (Figure 1): these data, collected across a global range of geographical locations (Figure S1), form the basis of the analyses presented here.

**Figure 3:**
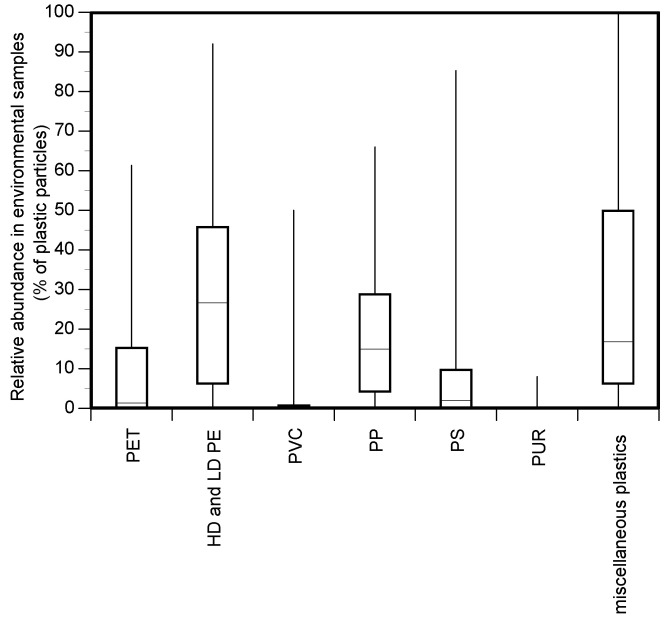
Variation (range (whisker), 25%ile–75%ile (box), and median (horizontal line)) in the relative abundance of polymers (% of particles) identified by studies of microplastics in freshwaters and estuaries.

There was considerable variation in the types of polymers and co-polymers identified by the various studies. A total of 69 polymers/co-polymers were identified, including the main production polymers, as classified by Plastics Europe [2], PET, polyethylene (PE), PVC, PP, PS and PUR. The techniques used to characterise microplastics from environmental samples cannot discriminate between low- and high-density polyethylene; hence, these two polymers were grouped as PE. Under those polymers classified as miscellaneous plastics, the more frequent polymers/co-polymers included polyamide (PA: number of studies, n = 34), polymethyl methacrylate (PMMA: n = 18), bioplastics (cellulose derivatives: n = 18) and polyethylene-vinyl acetate (PEVA: n = 11). Miscellaneous plastics also included those particles that were identified as plastic but could not be conclusively attributed to a specific polymer type. Many polymers/co-polymers occurred at low frequency (i.e., were identified as present in few studies) with the majority only identified in a single study (Figure S2). As well as considerable variation in the polymer types identified amongst studies, there was wide variation in the relative abundance reported within the polymer types identified, with data positively skewed for all polymers (Figure 3)—where individual polymer types were identified they were typically present at low relative abundance.

**Figure 4:**
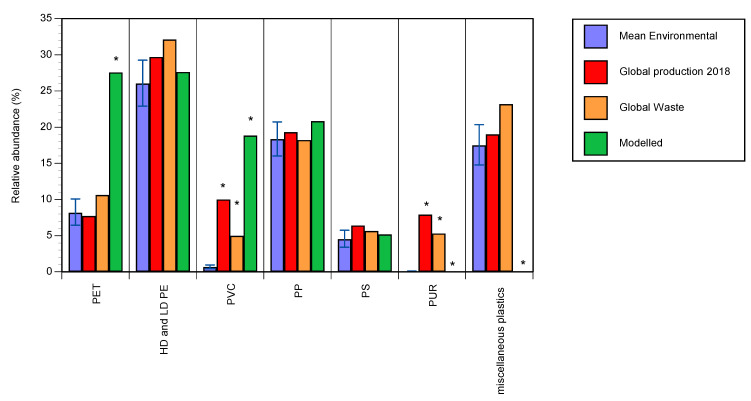
Relative abundance of polymers (mean % of particles ± SE) identified by studies of microplastics in freshwaters and estuaries compared with global production [2], estimated waste [9] and modelled release as microplastics to surface waters [3]. * Indicates a significant difference from environmental samples (*p* ≤ 0.05 Bonferroni corrected).

Despite the variation amongst studies, when compared with the relative proportions of polymers produced globally [2] and generated as waste [9], paired t-tests (using arcsine transformed data) indicated that microplastics comprised of PVC and PUR were significantly less frequent than would be expected (Figure 4). There was no significant difference in the relative abundance of PET, PE, PP, PS and miscellaneous plastics found in environmental samples when compared with the proportions expected from global production or generated as waste. Modelled estimates of the amount of microplastics released into the environment are typically based on the amount of product produced, with assumptions made on the rate of release [22,23,24]. Typically, these models provide estimates of the amount of microplastic entering the environment based on products, which cannot be matched to the microplastic particles found in environmental samples. To date, only one such model, that of Kawecki and Nowack [3], has provided polymer specific estimates of the amount of microplastics entering surface waters, which we presume is representative although the model is restricted to Switzerland. When compared with the Kawecki and Nowack model [3], PVC was considerably less abundant in environmental samples of microplastics (Figure 4). The Kawecki and Nowack model also overestimated the relative abundance of microplastics composed of PET. PUR and miscellaneous plastics were not included in the Kawecki and Nowack model [3] despite the latter comprising a large proportion of environmental microplastics (mean 17.5%). As the Kawecki and Nowack model is based only on data from Switzerland, further modelling using global data would provide additional information.

**Figure 5:**
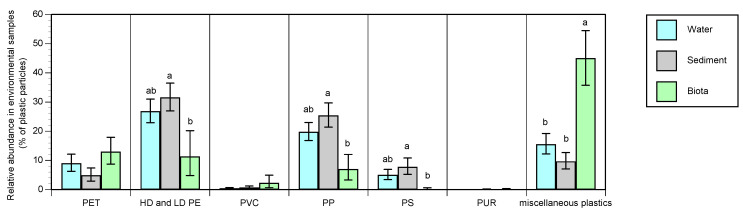
Mean relative abundance of polymers (% of particles ± SE) reported as microplastics in water (n = 39), sediment (n = 23) and biota (n = 10) sampled in freshwaters and estuaries. Influence of matrix (water, sediment or biota) *p* = 0.0362 from MANOVA. Significant differences within polymers were identified by Tukey’s test, where mean values that are not significantly different (within polymers) share the same letter.

When environmental samples of microplastics were separated by matrix, there was a significant difference in the relative frequency of polymers identified from samples of water, sediment and biota (identified by MANOVA, *p* = 0.0362), where the polymer profile of microplastics identified from biota was distinct from those of water and sediment (Figure 5). Differences within polymers, in terms of their relative abundance in water, sediment and biota, were identified using Tukey’s post hoc test (a more conservative statistical test than MANOVA). Despite the considerable variation in the data, PE, PP and PS were all significantly more frequent in sediment than biota, with water not significantly different to either sediment or biota, whereas miscellaneous plastics were significantly and substantially more frequent in biota than either water or sediment (Figure 5). PET, PVC and PUR were not significantly different among the three matrices, with the latter two polymers occurring at low relative abundance in all matrices. Further categorization of miscellaneous plastics indicated that PA was significantly more frequent in biota than water or sediment, but there was no difference in the relative abundance of either bioplastics or PMMA among the matrices, possibly due to the lower frequency of occurrence of these polymers (Figure S3).

## 4. Discussion

This study provides the first global analysis of the composition of microplastics found in freshwater and estuaries. A total of 69 different polymers and co-polymers were identified as present in the environment. However, most of these were found only in single studies. In addition to the wide array of different polymers identified, there was considerable variation between studies in the relative abundance of the more frequently observed polymers. Whilst such a variation may reflect local differences in source inputs, it is not possible at this stage to discount an influence of the methods used, which highlights the need for the consistency of methods between studies and a widely accepted standardized procedure to sample, extract and analyse microplastics.

Despite the considerable variation amongst different studies, our systematic analysis was able to identify trends in the occurrence of different polymers. Whilst the relative abundance of microplastics comprised of PET, PE, PP and PS in the environment was consistent with their relative global production, microplastics comprised of PVC and PUR were significantly less abundant than would be expected based on production [2] or generated as waste [9]. While PET, PE, PP and PS are widely used in the packaging of food and single use products, PVC and PUR are predominantly utilized in long lifespan products for the building and construction sector (Table 2). This mismatch could be due to various factors, such as a lower probability of PVC and PUR products being discarded into the environment, a lower likelihood of PVC and PUR products fragmenting in use or after being discarded to produce microplastics, or better recycling management of PVC- and PUR-based products.

A similar comparison of the microplastics found in freshwater and estuaries with the modelled proportions of polymers released to surface waters [3] showed that the relative abundances of PVC and PET in the environment have been overestimated. Furthermore, the model did not include PUR or miscellaneous plastics, despite these being important production polymers. The authors of the model [3] do highlight that processes involved in the transport and degradation of microplastics once released into the environment, which include fluvial transport, sedimentation and fragmentation of microplastics, were not accounted for in the model, but are likely to have a significant influence on the fate of polymers once they enter the environment. Nevertheless, our analysis indicates that models based on production, waste generated and assumed release do not predict the relative abundance of microplastic polymers in the environment accurately, and support the assertion of Kawecki and Nowack [3] that factors influencing the transport and fate of microplastics should be included in future models.

The results further indicate the importance of transport and fate for the polymer profile of microplastics in the environment. MANOVA indicated a significant difference among the matrices water, sediment and biota (Figure 5), despite relatively low replication and substantial variation in the data (both of which reduce the statistical power of the tests used). The profile of polymers in biota was most distinct from that of the microplastics in sediment, possibly suggesting that the biota studied (fish and bivalves) were more exposed to microplastics in water. However, given that the data available from biota were restricted to a limited number of species of fish and bivalves (and hence a limited number of feeding strategies), more data would be required, covering a wider range of taxa and feeding strategies, before a more definitive conclusion could be drawn. It was also apparent that, despite assumptions of the influence of density of polymers on transport and settlement [10] leading to density-dependent differences in their relative abundance in water and sediments, the partitioning of polymers between these matrices did not appear to follow differences in density (Figure 5 and Table 2 for density values). Whilst the density of particles influences the rate at which they sink, other factors must be involved in the deposition of microplastics and their incorporation into sediments. Further work on the fate of microplastics in the environment, and particularly the factors influencing their settlement behaviour, is required.

On the other hand, the data indicate that the relative abundance of miscellaneous plastics was significantly higher in biota than in sediments or in water. A closer look at the most frequently occurring components of this category highlights PA as being significantly more prevalent in biota (Figure S3). This finding could reflect active discrimination among microplastic particles by biota and subsequent uptake by ingestion, or it may reflect differential exposure of biota to microplastics, dependent upon where and how the animals feed [28,29,30]. Again, more data from a wider range of feeding strategies would be informative.

However, the relative abundance of the “others” category of miscellaneous plastics, comprising a wide variety of infrequently observed, lower production volume polymers and co-polymers, was also significantly higher in biota than in water or sediment (Figure S3), indicating that samples of microplastics extracted from freshwater and estuarine biota were more likely to be attributed to a polymer/co-polymer of this type. Whilst the relatively low number of reliable studies available on microplastics in biota may have influenced our findings, it is possible that the more aggressive separation techniques used to extract microplastics from animal tissue [31,32] affected the identification of polymers.

## 5. Conclusions

This systematic analysis of global data describing the polymer profile of microplastics in freshwaters and estuaries has highlighted the importance of careful analysis of the different polymers present in the environment when evaluating the impact of microplastics. The data available suggest that microplastics of PVC and PUR are less prevalent than expected and indicate that the polymer profile of microplastics in biota is distinct from that in the sediment and water, suggesting possible selectivity in the types of microplastics taken up by biota. Future studies and models need to consider processes influencing the transport and fate of microplastics—assumptions of partitioning of polymers between sediment and water, based on differences in the density of polymers do not appear to be supported by our findings. Despite these clear findings, the analyses were constrained by the limited number of studies available and inconsistencies in the methodologies used. There is an urgent need for consistency in the methods used for polymer extraction and identification, together with validation of these methodologies.

## Figures and Tables

**Table 1:** Search terms used to identify the evidence available on sampling and analytical methods used to characterise microplastics in freshwaters and estuaries.

Population	Intervention	Outcome
aggregate *	spectroscop *	count
colloid *	raman	quantif *
floc *	particle analysis	abundance
plankton *	pyrolysis	concentrate *
sediment *	sampl *	density
diet *	separat *	substance
content	identif *	state
* fibre	flotat *	morphology
* fiber	floatat *	dimension
* bead	microscop *	composition
fragment *	digest *	
pellet *	centrifuge *	
flake *	buoyan *	
nurdle		
dust		

* is a wildcard used to indicate any character string, to maximise search results.

**Table 2:** Characteristics and uses of polymers identified as microplastics in freshwaters and estuaries.

Polymer	Density (g cm^−3^) [25]	Global Production [2] (Million Tonnes)	Uses [26,27]
PET	1.37–1.38	27.6	Bottles for water, soft drinks, juices, cleaners, etc., food jars/pots, clothes, plastic films, microwavable packaging, etc.
PE	0.91–0.97	106.6	Reusable bags, trays and containers, agricultural film, food packaging film, toys, milk bottles, shampoo bottles, pipes, houseware, floor tiles, shower curtains, bubble wrap, wire insulation and electric cables, rubbish bags, chemical and detergent bottles, buckets, plants pots, outdoor furniture.
PVC	1.3–1.45	35.9	Window frames, profiles, floor and wall covering, pipes, cable insulation, garden hoses, inflatable pools, plumbing and guttering, doors, shower curtains, credit cards, synthetic leather, cosmetic containers, commercial cling wrap, packaging.
PP	0.89–0.92	69.3	Food packaging, sweet and snack, wrappers, hinged caps, microwave containers, pipes, automotive parts, bank notes, etc.
PS	0.96–1.05	23.0	Food packaging (dairy, fishery), building insulation, electrical and electronic equipment, inner liner for fridges, eyeglasses frames, foam packaging, food containers, take-out clamshells, plastic tableware, disposable cups, plates and cutlery, boxes for compact discs and cassettes, etc.
PUR	1.01–1.21	28.4	Building insulation, pillows and mattresses, insulating foams, surface coatings, rollers for printing, used in cars, carpet, flexible foam in furniture, etc.
PA	1.13–1.35	68.2	Fibres, bristles for toothbrushes, tubing, clothing, fishing line,
PMMA	1.17–1.20		Perspex, plexiglass, eyeglass lenses, touch screens, etc.
PEVA	0.93–0.95		Adhesive, foam padding, foam floats, ski boots, bicycle saddles, hockey pads, boxing gloves and helmets, wakeboards, fishing rods handles, etc.
Others			Hub caps (ABS); optical fibres (PBT); roofing sheets (PC); cable coating in telecommunications (PTFE); many others in clothing, aerospace, medical implants, surgical devices, membranes, valves and seals, protective coatings, etc.

## Supplementary Materials

Supplementary Materials can be found at https://www.mdpi.com/1660-4601/17/24/9304/s1. Figure S1: Map showing the geographical locations of the studies that were used for this review, Figure S2: Frequency of detection of polymers/co-polymers by studies of microplastics in water, sediment and biota sampled in freshwaters and estuaries; Figure S3: Mean relative abundance of polymers (% of particles ± SE), including more frequent miscellaneous plastics, reported as microplastics in water (n = 39), sediment (n = 23) and biota (n = 10) sampled in freshwaters and estuaries. Influence of matrix (water, sediment or biota) *p* = 0.044 from MANVOVA. Significant differences within polymers were identified by Tukey’s test, where means that are not significantly different (within polymers) share the same letter, Table S1: List of references used as evidence sources.

## Abbreviations

ABS	Acrylonitrile butadiene styrene
FTIR	Fourier Transform Infra-Red Spectroscopy
GCMS	Gas Chromatography - Mass Spectrometry
HDPE	High-density polyethylene
LDPE	Low-density polyethylene
MANOVA	Multivariate analysis of variance
PA	Polyamide
PBT	Polybutylene terephthalate
PC	Polycarbonates
PE	Polyethylene
PEVA	Polyethylene-vinyl acetate
PMMA	Polymethyl methacrylate
PP	Polypropylene
PS	Polystyrene
PTFE	Polytetrafluoroethylene
PU	Polyurethane
PVC	Polyvinyl chloride
