# Supplementary material for: Systematic Analysis of the Relative Abundance of Polymers Occurring as Microplastics in Freshwaters and Estuaries
Source: Int J Environ Res Public Health. 2020 Dec 12;17(24):9304. doi: 10.3390/ijerph17249304 (PMC7764371; doi:10.3390/ijerph17249304)
Supplement: Supplementary file 1 [file ijerph-17-09304-s001.pdf]

Electronic Supplementary Information

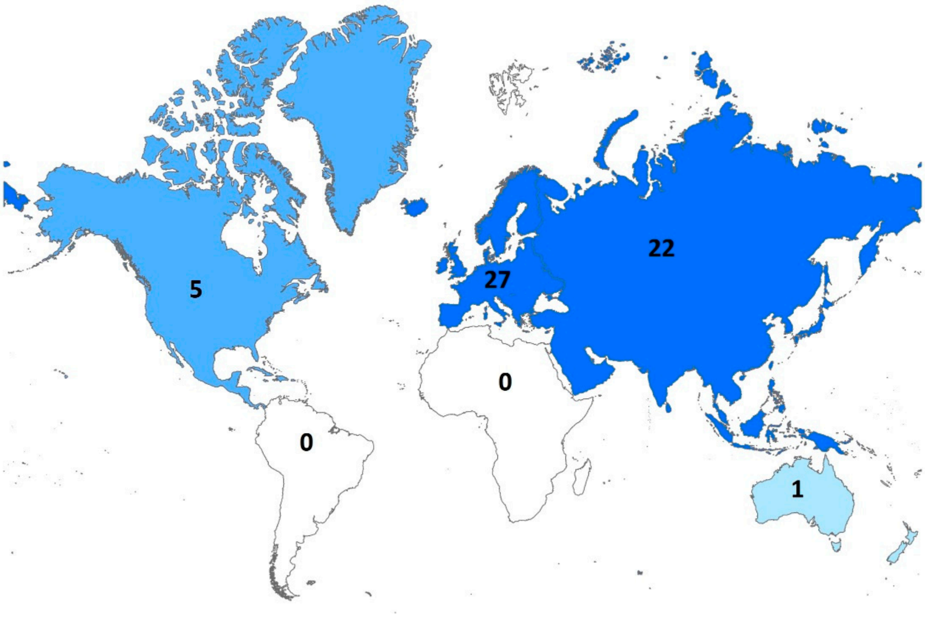

Figure S1. Map showing the geographical locations of the studies that were used for this review.

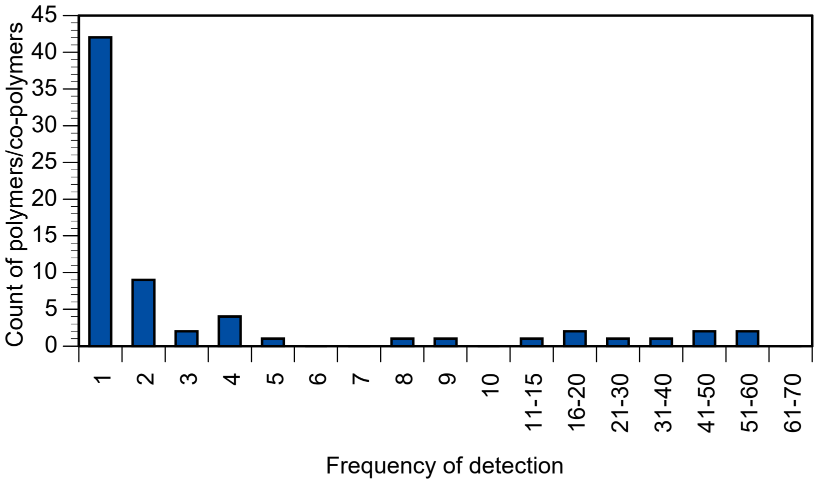

Figure S2. Frequency of detection of polymers/co-polymers by studies of microplastics in water, sediment and biota sampled in freshwaters and estuaries.

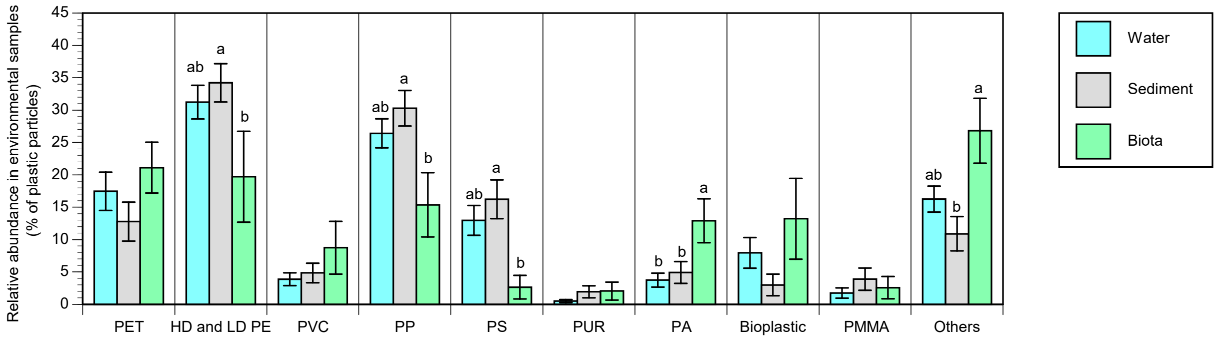

Figure S3. Mean relative abundance of polymers (% of particles ± SE) including more frequent miscellaneous plastics, reported as microplastics in water (n = 39), sediment (n = 23) and biota (n = 10) sampled in freshwaters and estuaries. Influence of matrix (water, sediment or biota) p = 0.0444 from MANVOVA. Significant differences within polymers were identified by Tukey's test, where means that are not significantly different (within polymers) share the same letter.

Table S1. List of references used as evidence sources.

1. Atwood, E.C.; Falcieri, F.M.; Piehl, S.; Bochow, M.; Matthies, M.; Franke, J.; Carniel, S.; Sclavo, M.; Laforsch, C.; Siegert, F. Coastal accumulation of microplastic particles emitted from the Po River, Northern Italy: Comparing remote sensing and hydrodynamic modelling with in situ sample collections. *Mar. Pollut. Bull.* **2019**, *138*, 561–574.
2. Ball, H., Cross, R., Grove, E., Horton, A., Johnson, A., Jurgens, M., Read, D., Svendsen, C. Sink To River – River To Tap. A Review of Potential Risks From Nanoparticles and Microplastics. Report, UK Water Industry Research Limited, 2019.
3. Ballent, A.; Corcoran, P.L.; Madden, O.; Helm, P.A.; Longstaffe, F.J. Sources and sinks of microplastics in Canadian Lake Ontario nearshore, tributary and beach sediments. *Mar. Pollut. Bull.* **2016**, *110*, 383–395.
4. Browne, M.A.; Crump, P.; Niven, S.J.; Teuten, E.; Tonkin, A.; Galloway, T.; Thompson, R. Accumulation of Microplastic on Shorelines Woldwide: Sources and Sinks. *Environ. Sci. Technol.* **2011**, *45*, 9175–9179.
5. Browne, M.A.; Galloway, T.S.; Thompson, R.C. Spatial patterns of plastic debris along estuarine shorelines. *Environ. Sci. Technol.* **2010**, *44*, 3404–3409.
6. Collard, F.; Gasperi, J.; Gilbert, B.; Eppe, G.; Azimi, S.; Rocher, V.; Tassin, B. Anthropogenic particles in the stomach contents and liver of the freshwater fish Squalius cephalus. *Sci. Total Environ.* **2018**, *643*, 1257–1264.
7. Cheung, L.T.O.; Lui, C.Y.; Fok, L. Microplastic contamination of wild and captive flathead grey mullet (Mugil cephalus). *Int. J. Environ. Res. Public Health* **2018**, *15*, 597-608.
8. de Lucia, G.A.; Vianello, A.; Camedda, A.; Vani, D.; Tomassetti, P.; Coppa, S.; Palazzo, L.; Amici, M.; Romanelli, G.; Zampetti, G.; et al. Sea Water Contamination in the Vicinity of the Italian Minor Islands Caused by Microplastic Pollution. *Water* **2018**, *10,* 1108-1121.
9. Dean, B.Y.; Corcoran, P.L.; Helm, P.A. Factors influencing microplastic abundances in nearshore, tributary and beach sediments along the Ontario shoreline of Lake Erie. *J. Great Lakes Res.* **2018**, *44*, 1002–1009.
10. Di, M.; Liu, X.; Wang, W.; Wang, J. Manuscript prepared for submission to environmental toxicology and pharmacology pollution in drinking water source areas: Microplastics in the Danjiangkou Reservoir, China. *Environ. Toxicol. Pharmacol.* **2019**, *65*, 82–89.
11. Dris, R. First assessement of sources and fate of macro and micro plastics in urban hydrosystems : Case of Paris megacity PhD thesis, Universit´e Paris-Est, France, 2017.
12. Frère, L.; Paul-Pont, I.; Moreau, J.; Soudant, P.; Lambert, C.; Huvet, A.; Rinnert, E. A semi-automated Raman micro-spectroscopy method for morphological and chemical characterizations of microplastic litter. *Mar. Pollut. Bull.* **2016**, *113*, 461–468.

13. Frère, L.; Paul-Pont, I.; Rinnert, E.; Petton, S.; Jaffré, J.; Bihannic, I.; Soudant, P.; Lambert, C.; Huvet, A. Influence of environmental and anthropogenic factors on the composition, concentration and spatial distribution of microplastics: A case study of the Bay of Brest (Brittany, France). *Environ. Pollut.* **2017**, *225*, 211–222.
14. Garaba, S.P.; Dierssen, H.M. An airborne remote sensing case study of synthetic hydrocarbon detection using short wave infrared absorption features identified from marine-harvested macro- and microplastics. *Remote Sens. Environ*. **2018**, *205*, 224–235.
15. Gündoğdu, S.; Çevik, C.; Ayat, B.; Aydoğan, B.; Karaca, S. How microplastics quantities increase with flood events? An example from Mersin Bay NE Levantine coast of Turkey. *Environ. Pollut*. **2018**, *239*, 342–350.
16. Hendrickson, E.M. Microplastics in the surface water and sediments of western Lake Superior as determined via microscopy, Pyr-GC/MS, and FTIR. Master Thesis, University of Minnesota, USA, 2017.
17. Horton, A.A.; Svendsen, C.; Williams, R.J.; Spurgeon, D.J.; Lahive, E. Large microplastic particles in sediments of tributaries of the River Thames, UK – Abundance, sources and methods for effective quantification. *Mar. Pollut. Bull.* **2017**, *114*, 218–226.
18. Imhof, H.K.; Ivleva, N.P.; Schmid, J.; Niessner, R.; Laforsch, C. Contamination of beach sediments of a subalpine lake with microplastic particles. *Curr. Biol.* **2013**, *23*, R867-R868.
19. Jabeen, K.; Su, L.; Li, J.; Yang, D.; Tong, C.; Mu, J.; Shi, H. Microplastics and mesoplastics in fish from coastal and fresh waters of China. *Environ. Pollut*. **2017**, *221*, 141–149.
20. Jiang, Y.; Zhao, Y.; Wang, X.; Yang, F.; Chen, M.; Wang, J. Characterization of microplastics in the surface seawater of the South Yellow Sea as affected by season. *Sci. Total Environ*. **2020**, *724*, 138375.
21. Kataoka, T.; Nihei, Y.; Kudou, K.; Hinata, H. Assessment of the sources and inflow processes of microplastics in the river environments of Japan. *Environ. Pollut.* **2019**, *244*, 958–965.
22. Kazour, M.; Jemaa, S.; El Rakwe, M.; Duflos, G.; Hermabassiere, L.; Dehaut, A.; Le Bihanic, F.; Cachot, J.; Cornille, V.; Rabhi, K.; et al. Juvenile fish caging as a tool for assessing microplastics contamination in estuarine fish nursery grounds. *Environ. Sci. Pollut. Res.* **2020**, *27*, 3548–3559.
23. Lahens, L.; Strady, E.; Kieu-Le, T.C.; Dris, R.; Boukerma, K.; Rinnert, E.; Gasperi, J.; Tassin, B. Macroplastic and microplastic contamination assessment of a tropical river (Saigon River, Vietnam) transversed by a developing megacity. *Environ. Pollut.* **2018**, *236*, 661–671.
24. Li, R.; Zhang, L.; Xue, B.; Wang, Y. Abundance and characteristics of microplastics in the mangrove sediment of the semi-enclosed Maowei Sea of the south China sea: New implications for location, rhizosphere, and sediment compositions. *Environ. Pollut*. **2019**, *244*, 685–692.
25. Long, Z.; Pan, Z.; Wang, W.; Ren, J.; Yu, X.; Lin, L.; Lin, H.; Chen, H.; Jin, X. Microplastic abundance, characteristics, and removal in wastewater treatment plants in a coastal city of China. *Water Res*. **2019**, *155*, 255–265.
26. Luo, W.; Su, L.; Craig, N.J.; Du, F.; Wu, C.; Shi, H. Comparison of microplastic pollution in different water bodies from urban creeks to coastal waters. *Environ. Pollut.* **2019**, *246*, 174–182.
27. Lusher, A.L., Nina T. Buenaventura, David P. Eidsvoll, Jan-Erik Thrane, A.Ø. and M.J. Freshwater microplastics in Norway A first look at sediment, biota and historical plankton samples from Lake Mjøsa and Lake Femunden. Report, NIVA 7326-2018, 2018.
28. Majewsky, M.; Bitter, H.; Eiche, E.; Horn, H. Determination of microplastic polyethylene (PE) and polypropylene (PP) in environmental samples using thermal analysis (TGA-DSC). *Sci. Total Environ.* **2016**, *568*, 507–511.
29. Mani, T.; Blarer, P.; Storck, F.R.; Pittroff, M.; Wernicke, T.; Burkhardt-Holm, P. Repeated detection of polystyrene microbeads in the lower Rhine River. *Environ. Pollut*. **2019**, *245*, 634–641.
30. McGoran, A.R.; Cowie, P.R.; Clark, P.F.; McEvoy, J.P.; Morritt, D. Ingestion of plastic by fish: A comparison of Thames Estuary and Firth of Clyde populations. *Mar. Pollut. Bull*. **2018**, *137*, 12–23.
31. Peng, G.; Xu, P.; Zhu, B.; Bai, M.; Li, D. Microplastics in freshwater river sediments in Shanghai, China: A case study of risk assessment in mega-cities. *Environ. Pollut.* **2018**, *234*, 448–456.

32. Peters, C.A.; Hendrickson, E.; Minor, E.C.; Schreiner, K.; Halbur, J.; Bratton, S.P. Pyr-GC/MS analysis of microplastics extracted from the stomach content of benthivore fish from the Texas Gulf Coast. *Mar. Pollut. Bull.* **2018**, *137*, 91–95.
33. Pivokonsky, M.; Cermakova, L.; Novotna, K.; Peer, P.; Cajthaml, T.; Janda, V. Occurrence of microplastics in raw and treated drinking water. *Sci. Total Environ*. **2018**, *643*, 1644–1651.
34. Rodrigues, M.O.; Abrantes, N.; Gonçalves, F.J.M.; Nogueira, H.; Marques, J.C.; Gonçalves, A.M.M. Spatial and temporal distribution of microplastics in water and sediments of a freshwater system (Antuã River, Portugal). *Sci. Total Environ*. **2018**, *633*, 1549–1559.
35. Sadri, S.S. Investigation of microplastic debris in marine surface waters using different sampling methods. PhD Thesis, University of Plymouth, UK, 2015.
36. Santillo, D., Brigden, K., Pasteur, V., Nicholls, F., Morozzo, P., Johnston, P. Plastic pollution in UK's rivers: a "snapshot" survey of macro-and microplastic contamination in surface waters of 13 river systems across England, Wales, Scotland and Northern Ireland. Technical Report, Greenpeace Research Laboratories, 2019.
37. Sighicelli, M.; Pietrelli, L.; Lecce, F.; Iannilli, V.; Falconieri, M.; Coscia, L.; Di Vito, S.; Nuglio, S.; Zampetti, G. Microplastic pollution in the surface waters of Italian Subalpine Lakes. *Environ. Pollut*. **2018**, *236*, 645–651.
38. Su, L.; Cai, H.; Kolandhasamy, P.; Wu, C.; Rochman, C.M.; Shi, H. Using the Asian clam as an indicator of microplastic pollution in freshwater ecosystems. *Environ. Pollut*. **2018**, *234*, 347–355.
39. Talvitie, J.; Mikola, A.; Setälä, O.; Heinonen, M.; Koistinen, A. How well is microlitter purified from wastewater? – A detailed study on the stepwise removal of microlitter in a tertiary level wastewater treatment plant. *Water Res*. **2017**, *109*.
40. Talvitie, J.; Mikola, A.; Koistinen, A.; Setälä, O. Solutions to microplastic pollution – Removal of microplastics from wastewater effluent with advanced wastewater treatment technologies. *Water Res.* **2017**, *123*, 401–407.
41. Tan, X.; Yu, X.; Cai, L.; Wang, J.; Peng, J. Microplastics and associated PAHs in surface water from the Feilaixia Reservoir in the Beijiang River, China. *Chemosphere* **2019**, *221*, 834–840.
42. van der Wal, M.; van der Meulen, M.; Tweehuijsen, G.; Peterlin, M.; Palatinus, A.; Kovač Viršek, M.; Coscia, L.; Kržan, A. SFRA0025: Identification and Assessment of Riverine Input of (Marine) Litter; Report, Eunomia Research & Consulting Ltd, 2015.
43. Veerasingam, S.; Mugilarasan, M.; Venkatachalapathy, R.; Vethamony, P. Influence of 2015 flood on the distribution and occurrence of microplastic pellets along the Chennai coast, India. *Mar. Pollut. Bull*. **2016**, *109*, 196–204.
44. Vianello, A.; Boldrin, A.; Guerriero, P.; Moschino, V.; Rella, R.; Sturaro, A.; Da Ros, L. Microplastic particles in sediments of Lagoon of Venice, Italy: First observations on occurrence, spatial patterns and identification. *Estuar. Coast. Shelf Sci*. **2013**, *130*, 54–61.
45. Wang, J.; Peng, J.; Tan, Z.; Gao, Y.; Zhan, Z.; Chen, Q.; Cai, L. Microplastics in the surface sediments from the Beijiang River littoral zone: Composition, abundance, surface textures and interaction with heavy metals. *Chemosphere* **2017**, *171*, 248–258.
46. Xiong, X.; Zhang, K.; Chen, X.; Shi, H.; Luo, Z.; Wu, C. Sources and distribution of microplastics in China's largest inland lake – Qinghai Lake. *Environ. Pollut*. **2018**, *235*, 899–906.
47. Xiong, X.; Wu, C.; Elser, J.J.; Mei, Z.; Hao, Y. Occurrence and fate of microplastic debris in middle and lower reaches of the Yangtze River – From inland to the sea. *Sci. Total Environ*. **2019**, *659*, 66–73.
48. Yan, M.; Nie, H.; Xu, K.; He, Y.; Hu, Y.; Huang, Y.; Wang, J. Microplastic abundance, distribution and composition in the Pearl River along Guangzhou city and Pearl River estuary, China. *Chemosphere* **2019**, 217, 879–886.
49. Yang, L.; Li, K.; Cui, S.; Kang, Y.; An, L.; Lei, K. Removal of microplastics in municipal sewage from China's largest water reclamation plant. *Water Res*. **2019**, *155*, 175–181.
50. Zada, L.; Leslie, H.A.; Vethaak, A.D.; Tinnevelt, G.H.; Jansen, J.J.; de Boer, J.F.; Ariese, F. Fast microplastics identification with stimulated Raman scattering microscopy. *J. Raman Spectrosc.* **2018**, *49*, 1136–1144.

51. Zhang, K.; Xiong, X.; Hu, H.; Wu, C.; Bi, Y.; Wu, Y.; Zhou, B.; Lam, P.K.S.; Liu, J. Occurrence and Characteristics of Microplastic Pollution in Xiangxi Bay of Three Gorges Reservoir, China. *Environ. Sci. Technol*. **2017**, *51*, 3794–3801.
52. Zhang, K.; Su, J.; Xiong, X.; Wu, X.; Wu, C.; Liu, J. Microplastic pollution of lakeshore sediments from remote lakes in Tibet plateau, China. *Environ. Pollut*. **2016**, *219*, 450–455.
53. Zhang, K.; Gong, W.; Lv, J.; Xiong, X.; Wu, C. Accumulation of floating microplastics behind the Three Gorges Dam. *Environ. Pollut*. **2015**, *204*, 117–123.
54. Zhang, X.; Leng, Y.; Liu, X.; Huang, K.; Wang, J. Microplastics' Pollution and Risk Assessment in an Urban River: A Case Study in the Yongjiang River, Nanning City, South China. *Expo. Heal*. **2020**, *12*, 141–151.
55. Zobkov, M.B.; Esiukova, E.E.; Zyubin, A.Y.; Samusev, I.G. Microplastic content variation in water column: The observations employing a novel sampling tool in stratified Baltic Sea. *Mar. Pollut. Bull*. **2019**, *138*, 193–205.
